# Predictors for pulmonary artery involvement in Takayasu arteritis and its cluster analysis

**DOI:** 10.1186/s13075-022-02987-4

**Published:** 2023-01-14

**Authors:** Hua Liao, Nan Zhang, Lili Pan, Juan Du, Jiayi Liu, Yi Zheng

**Affiliations:** 1 Departments of Rheumatology and Immunology, Beijing Anzhen Hospital, Capital Medical University, Beijing, China; 2 Departments of Rheumatology, Beijing Chaoyang Hospital, Capital Medical University, #8 Gong-Ti South Road, Chaoyang District, Beijing, 100020 China

**Keywords:** Takayasu arteritis, Pulmonary artery, Cluster analysis

## Abstract

**Objective:**

To investigate the clinical characteristics and the site of pulmonary involvement in Takayasu arteritis (TAK) patients with pulmonary artery involvement (PAI).

**Methods:**

We retrospectively investigated data of 141 TAK patients. The clinical and image data of the patients with and without PAI were analyzed and compared. The patients were followed up. The major outcome was all-cause mortality. The minor outcome was exacerbation or new occurrence of PAI, which leads to disease progression events.

**Results:**

For the 141 TAK patients considered, PAI was detected in 65 (46.1%) patients. TAK patients with PAI had a significantly higher cumulative incidence of events than those without PAI (*P* < 0.001). The frequencies of the following were significantly higher in TAK with PAI than those in TAK without PAI: disease duration [median 96 months (IQR: 24–174) vs. median 42 months (IQR: 6–120); *P* = 0.012], hemoptysis (10.8% vs. 1.32%; *P* = 0.040), oppression in the chest (40.0% vs. 21.1%; *P* = 0.014), fever (23.1% vs. 9.21%; *P* = 0.024), *Mycobacterium tuberculosis* infection (21.5% vs. 6.57%; *P* = 0.010), pulmonary hypertension (PAH) (21.5% vs. 2.6%; *P* < 0.001), pulmonary infarction (41.5% vs. 0*%; P* < 0.001), and hypoxemia (18.5% vs. 1.3%; *P* < 0.001). Multivariate logistic regression analysis of data of TAK patients with symptom presentation showed that oppression in the chest (OR: 2.304; 95% CI: 1.024–5.183; *P* = 0.044) and thoracic aorta involvement (OR: 2.819; 95% CI: 1.165–6.833; *P* = 0.022) were associated with PAI. The cluster analysis performed for data of TAK patients with PAI revealed that the cluster characterized as the upper lobe of the right lung (Cluster1) had the worst prognosis.

**Conclusion:**

In TAK, PAI is associated with thoracic aorta involvement. In TAK patients with PAI, the involvement of the upper lobe of the right lung is characterized with the worst prognosis.

## Introduction

Takayasu arteritis (TAK) is a chronic, large-vessel vasculitis that mostly affects young Asian women [1–4]. Vascular inflammation in TAK primarily invades the aorta and its major branches and often leads to arterial stenosis, occlusion and aneurysms, which in turn cause tissue/organ ischemia/dysfunction and eventually lead to death [3]. The pulmonary artery (PA) is one of the major arteries involved in TAK patients. Pulmonary artery involvement (PAI) is frequent and, according to previous reports [5–7], occurs in 6.9–86% of patients with TAK. Isolated PAI in TAK rarely happens [8] and PAI is normally accompanied with damage to aorta and other large arteries [9]. Stenosis, occlusion, or embolism of the PA can lead to perfusion defects, pulmonary infarction, and pulmonary arterial hypertension (PAH), which may result in heart failure [10, 11]. Thus, PAI has a direct adverse effect on the prognosis of TAK patients. However, PAI in TAK is very insidious, and its detection in most of the patients only happens because of serious complications, such as pulmonary infarction, pulmonary hypertension, and hypoxemia. In such situations, unfortunately, the PA disease has already progressed to severe stenosis and occlusion, and the optimal timing of drug treatment has been missed. Thus, early identification of PAI is paramount for prolonging the survival time of the patients and improving their quality of life by preventing disease progression.

In the present study, we aimed to investigate the clinical characteristics, the inflammatory cytokines, and the site of the pulmonary involvement in TAK patients with PAI by using computer tomography pulmonary angiography (CTPA) and by performing cluster analysis for prognostic evaluation to facilitate early identification of PAI, prolong patient survival, and improve patient’s quality of life.

## Methods

### Patients

A retrospective study was performed. The study cohort consisted of 193 patients with TAK from January 2013 to June 2021 from inpatient services in Beijing Anzhen Hospital, Capital Medical University, Beijing, China. All TAK patients fulfilled the criteria of the 1990 American College of Rheumatology (ACR) for TAK [1]. Among them, fifty-two patients did not receive CTPA and 141 patients received CTPA. In our final analysis, we studied 141 patients with CTPA. The disease duration ranged from 1 to 636 months, with a median course of 48 months. Disease activity was evaluated according to the National Institutes of Health (NIH) criteria [12]. TAK vascular involvement was classified using Numano classification criteria [13] by examining the imaging data.

### Data collection

Laboratory parameters and clinical data were obtained from the medical records. Clinical characteristics, including the disease duration, complications, and medication history, were collected for all patients, and the NIH scores were recorded.

The tests were carried out at the diagnosis of Takayasu’s arteritis. Blood samples (6 mL each) were collected from each patient for laboratory measurements. Clinical data, including hepatic and renal function, were collected for all patients by using an automatic biochemical analyzer (7600-120, Hitachi, Tokyo, Japan). Serum immune globulin and complement were measured by using an automatic biochemical analyzer (AU5400, Beckman Coulter, CA, USA). The serum C-reaction protein concentrations were determined by immunonephelometry, and the ESR was measured by the traditional Westergren method. IL-6 levels were tested by using chemiluminescent immunoassay techniques (Beckman Coulter, CA, USA).

All patients had either a vascular computed tomography angiography (CTA) and/or a magnetic resonance angiography (MRA) of the aorta and of all of its primary branches at presentation. Vascular Doppler data were additionally used to evaluate the involvement of the peripheral arteries, such as the axillary and humeral arteries, which could not always be visualized with CTA angiography and MRA. The vascular Doppler specialist performed peripheral vascular Doppler examinations. CTA and MRA were interpreted in consensus by 2 radiologists. The arterials of the patients were evaluated for the presence or absence of any of the following vascular lesions: wall thickening (wall thickness > 2 mm), stenosis [defined as a more than 25% (>25%) decrease in the diameter of the diseased artery when compared with the normal PA], dilatation [defined as a more than 25% (>25%), but a less than 50% (<50%), increase in the diameter of the diseased artery when compared with the normal PA], aneurysm [defined as a more than 50% (>50%) increase in the diameter of the diseased artery when compared with the normal PA], and occlusion. The presence of any of these lesions in a given vessel during the follow-up period was recorded as the involvement of that vessel.

The retrospective study was conducted in accordance with the Declaration of Helsinki and was approved by the Ethics Committee of Beijing Anzhen Hospital, Capital Medical University [No. 2022173X].

### Follow-up

One hundred forty-one patients were followed up and the major outcome and the minor outcome were recorded. The major outcome was all-cause mortality. The minor outcome was the combined secondary endpoints, which was exacerbation or new occurrence of PAI and disease progression events. For evaluating exacerbation or new occurrence of PAI, the changes in pulmonary arteries that concerned wall thickening, stenosis, occlusion, and dilatation were assessed by CTPA. Exacerbation or new PAI lead to disease progression events including exacerbation or new occurrence of pulmonary hypertension, exacerbation or new onset of hypoxemia, new pulmonary infarction, exacerbation or new onset of chest pain, and death.

### Cluster analysis

CTPA data from the 65 TAK patients identified to have PAI were retrospectively recorded. For our statistical analysis, we used R software and Ward’s method of hierarchical clustering for clustering analysis of the following vascular beds: the main PA, left PA, right PA, right upper lobar PA, apical segment, posterior segment, anterior segment, right middle lobar PA, medial segment, lateral segment, right lower lobar PA, superior segment, medial basal segment, anterior basal segment, lateral basal segment, posterior basal segment, left upper lobar PA, apicoposterior segment, anterior segment, superior lingular segment, inferior lingular segment, left lower lobar PA, superior segment, anteromedial segment, lateral basal segment, and posterior basal segment.

### Statistical analysis

Continuous data were expressed as mean ± standard deviation (mean ± SD, $\overline{x}\pm s$), and the independent-samples *t*-test was used to compare differences between two groups. Medians and interquartile ranges were used to describe abnormally distributed data, and the rank-sum test was used to compare differences between two groups. Discrete variables were described through frequencies and percentages. Depending on the number of events, chi-square or Fisher’s exact test was performed. To identify predictors of TAK-PAI, univariate and multivariate logistic regression analyses were performed. Odds ratio (OR) and 95% confidence interval (CI) were calculated. The cumulative incidence of disease progression events was calculated by the Kaplan-Meier method. All statistical analyses were performed using the Statistical Package for Social Sciences version 22.0 (SPSS, Chicago, IL, USA). *P* < 0.05 was considered statistically significant.

## Results

### Basic characteristics of TAK patients with PAI

According to CTPA, PA was involved in 65 patients (patients with PAI) and was not involved in 76 patients (patients without PAI). One hundred forty-one TAK patients were included. Patients’ clinical characteristics, laboratory findings, and the artery involvement are presented in Table 1. TAK with PAI was detected in 65 (46.1%) patients. The mean age of patients with TAK with PAI was 39 ± 12 years, which did not differ from patients without PAI. TAK with PAI had a longer disease duration than TAK without PAI [median 96 months (IQR: 24–174) vs. median 42 months (IQR: 6–120); *P* = 0.012, respectively]. In patients with TAK with PAI, hemoptysis (10.8% vs. 1.32%; *P* = 0.040), oppression in the chest (40.0% vs. 21.1%; *P* = 0.014), fever (23.1% vs. 9.21%; *P* = 0.024), and *Mycobacterium tuberculosis* infection (including tuberculosis and pleural tuberculosis) (21.5% vs. 6.57%; *P* = 0.010) were significantly more frequent compared with those in patients with TAK without PAI.

**Table 1:** Baseline characteristics

	TAK with PAI (*n*=65)	TAK without PAI (*n*=76)	*P* value
**Clinical characteristics**			
Disease duration (months)	96 (24,174)	42 (6120)	0.012
Female sex, *n* (%)	62 (95.4)	70 (92.1)	0.654
Age (years)	39 ± 12	38 ± 12	0.447
Age of onset (years)	29 ± 11	31 ± 12	0.662
BMI	22.90 ± 3.78	21.81 ± 2.84	0.116
**Numano subtype**			
Type I, *n* (%)	5 (7.69)	11 (14.5)	0.206
Type IIa, *n* (%)	5 (7.69)	2 (2.63)	0.322
Type IIb, *n* (%)	13 (20.0)	12 (15.8)	0.514
Type III, *n* (%)	1 (1.54)	5 (6.58)	0.289
Type IV, *n* (%)	0 (0)	5 (6.58)	0.099
Type V, *n* (%)	41 (63.1)	41 (53.9)	0.273
**Symptoms**			
Hemoptysis, *n* (%)	7 (10.8)	1 (1.32)	0.040
Oppression in the chest, *n* (%)	26 (40.0)	16 (21.1)	0.014
Fever, *n* (%)	15 (23.1)	7 (9.21)	0.024
Dizziness, *n* (%)	30 (46.2)	38 (50.0)	0.649
Palpitations, *n* (%)	10 (15.4)	10 (13.1)	0.706
Chest pain, *n* (%)	15 (23.1)	16 (21.1)	0.772
**Comorbidities and personal history**			
*Mycobacterium tuberculosis* infection, *n* (%)	14 (21.5)	5 (6.57)	0.010
PAH, *n* (%)	14 (21.5)	2 (2.6)	<0.001
Pulmonary infarction, *n* (%)	27 (41.5)	0 (0)	<0.001
Hypoxemia, *n* (%)	12 (18.5)	1 (1.3)	<0.001
Hypertension, *n* (%)	19 (29.2)	26 (34.2)	0.527
Diabetes mellitus, *n* (%)	3 (4.62)	6 (7.89)	0.654
Hyperlipidemia, *n* (%)	6 (9.23)	13 (17.1)	0.172
Coronary heart disease, *n* (%)	9 (13.8)	5 (6.58)	0.150
Cerebrovascular disease, *n* (%)	2 (3.08)	6 (7.89)	0.386
History of smoking, *n* (%)	3 (4.62)	7 (9.21)	0.465
History of alcohol, *n* (%)	1 (1.54)	5 (6.58)	0.289
**Blood biochemistry, inflammatory markers, and disease activity**			
WBC (10^9^/L)	7.4 ± 2.5	7.1 ± 2.6	0.484
HGB (10^9^/L)	120 ± 20	120 ± 17	0.957
PLT (10^9^/L)	283 ± 96	269 ± 86	0.795
ESR (mm/1h)	17 (7, 36)	17 (10, 30)	0.826
CRP (mg/l)	5.96 (1.14, 19.86)	3.03(0.59, 8.29)	0.056
NIH score	2 (2, 3)	2 (2, 3)	0.330
**The artery involvement**			
Common carotid artery, *n* (%)	55 (84.6)	55 (72.4)	0.080
Vertebral artery, *n* (%)	23 (35.4)	23 (30.3)	0.518
Arteria subclavia, *n* (%)	58 (89.2)	60 (78.9)	0.099
Superior mesenteric artery, *n* (%)	18 (27.7)	20 (26.3)	0.854
Renal artery, *n* (%)	18 (27.7)	31 (40.8)	0.104
Common iliac artery, *n* (%)	9( 13.8)	13 (17.1)	0.595
Thoracic aorta, *n* (%)	56 (86.2)	45 (59.2)	<0.001
Aorta abdominalis, *n* (%)	34 (52.3)	41 (53.9)	0.846

*TAK* Takayasu’s arteritis, *PAI* pulmonary artery involvement, *PAH* pulmonary arterial hypertension, *WBC* white blood cell, *HGB* hemoglobin, *PLT* platelet count, *ESR* erythrocyte sedimentation rate, *CRP* C-reactive protein, *NIH* National Institutes of Health

TAK patients with PAI, compared to those without PAI, had a higher risk of PAH [21.5% vs. 2.6%; *P* < 0.001], pulmonary infarction (41.5% vs. 0%; *P* < 0.001), and hypoxemia (18.5% vs. 1.3%: *P* < 0.001). There were two cases of PAH in TAK patients without PAI. In one case, right heart catheterization proved the presence of PAH of left heart origin. In another case, echocardiography suggested PAH, but the patient refused right heart catheterization. The incidence of Numano type V was higher in TAK patients with PAI. No significant differences were found with respect to gender, age, total white blood cell (WBC), hemoglobin (HGB), platelet (PLT), erythrocyte sedimentation rate (ESR), C-reaction protein (CRP), and NIH score between the patients of TAK with PAI and those without PAI. Echocardiography, arterial ultrasound, and MRA/CTA were performed in all the TAK patients. The incidence of thoracic aorta involvement in patients with PAI was significantly higher than that in patients without PAI (*P* < 0.001).

### Related factors of TAK patients with PAI

Multivariate logistic regression analysis based on baseline characteristics revealed that for TAK patients with symptom presentation, oppression in the chest (OR: 2.304; 95% CI: 1.024–5.183; *P* = 0.044) and thoracic aorta involvement (OR: 2.819; 95% CI: 1.165–6.822; *P* = 0.022) were associated with the occurrence of PAI (Table 2).

**Table 2:** Predictors of TAK with PAI

	Univariate analysis		Multivariate analysis	
	OR (95.0%CI)	*P* value	OR (95.0%CI)	*P* value
Disease duration (months)	1.002 (0.999–1.005)	.136		
oppression in the chest	2.500 (1.191–5.250)	.015	2.304 (1.024–5.183)	.044
Hemoptysis	9.052 (1.083–75.65)	.042	5.021 (0.561–44.95)	.149
Fever	2.957 (1.123–7.787)	.028	2.509 (0.867–7.261)	.090
*Mycobacterium tuberculosis* infection	3.898 (1.320–11.51)	.014	2.223 (0.697–7.068)	.177
Thoracic aorta involvement	4.286 (1.851–9.924)	.001	2.819 (1.165–6.822)	.022

*TAK* Takayasu’s arteritis, *PAI* pulmonary artery involvement

### Imaging features of PA in 65 TAK patients with PAI

**Fig. 1:**
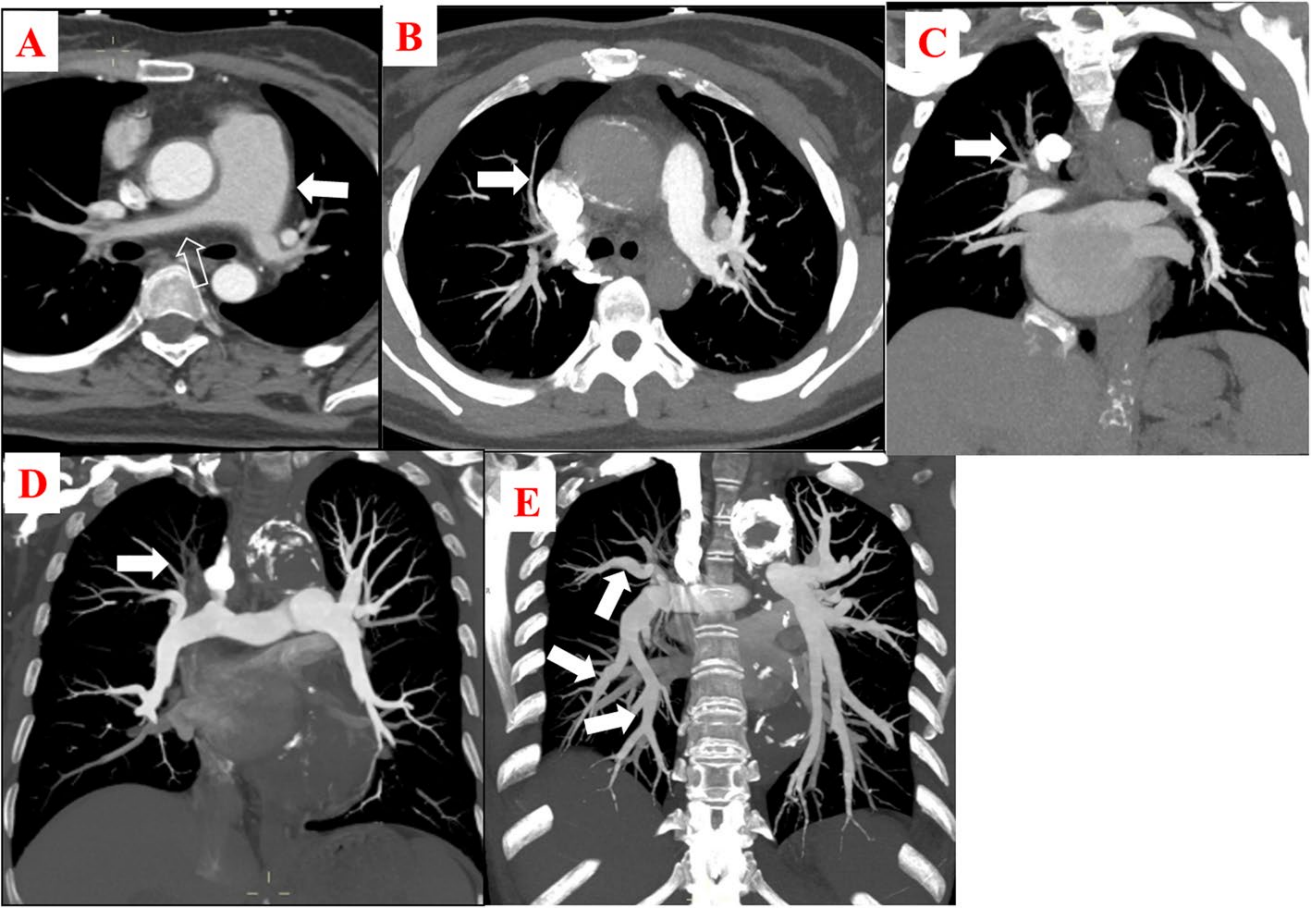
Representative pulmonary artery involvement images. The main pulmonary artery widened and vessel wall thickness (white arrows in **A**). The right main pulmonary artery vessel wall thickness and stenosis (white empty arrows in **A**). Stenosis of the right upper pulmonary artery (white arrows in **B**). Occlusion of the right upper pulmonary artery (white arrows in **C**). Occlusion of the right main pulmonary artery (white arrows in **D**). Dilation of the pulmonary artery (white arrows in **E**)

The involved PA sites according to CTPA were recorded, which included main PA, lobar PA, and segmental PA. The lesions of each site were classified into thickening, stenosis, occlusion, dilation, and aneurysm. Representative images of PAI are presented in Fig. 1. The main PA lesions mainly included thickening and stenosis. Lobar PA lesions mainly included stenosis and occlusion. Occlusion was dominant in segmental PA. It should be added that occlusion was the most common lesion, which occurred in 188 (49.74%) instances, followed by stenosis, which happened in 137 (36.24%) instances; this information is included in Table 3.

**Table 3:** The damage of PAI in TAK

Group	Main PA, *n* (%)	Lobar PA, *n* (%)	Segment PA, *n* (%)	Sum, *n* (%)
Thickening	36 (9.52)	3 (0.79)	7 (1.85)	46 (12.17)
Stenosis	33 (8.73)	18 (4.76)	86 (22.75)	137 (36.24)
Occlusion	5 (1.32)	30 (7.94)	153 (40.48)	188 (49.74)
Dilation	1 (0.26)	5 (1.32)	0 (0)	6 (1.59)
Aneurysm	0 (0)	0 (0)	1 (0.26)	1 (0.26)

*PA* pulmonary artery, *TAK* Takayasu’s arteritis, *PAI* pulmonary artery involvement

**Fig. 2:**
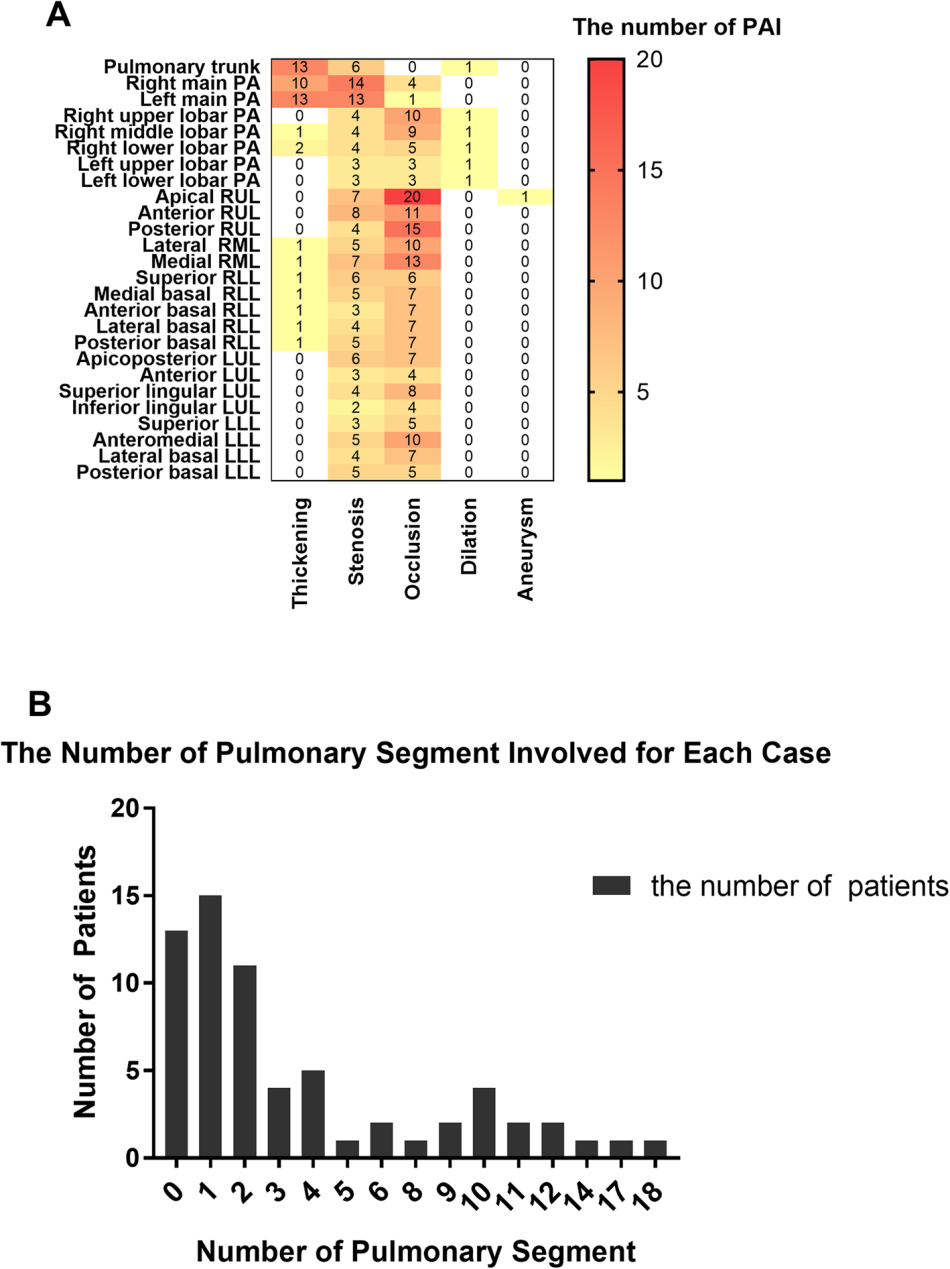
Distribution of pulmonary artery involvement in patients with Takayasu arteritis. **A** The heat map depicting the number of main pulmonary artery, lobar pulmonary artery and segment pulmonary artery. **B** The number of pulmonary segments involved for each case. The *X*-axis is the number of affected pulmonary artery segments, and the *Y*-axis is the number of affected patients with Takayasu arteritis

Sixty-five TAK patients with PAI had 378 PA lesions (see Fig. 2A). Bilateral affected area was found in 27 patients and unilateral affected area was identified in 36 patients. There were 46 thickening sites (12.2%), mainly in the main PA (13; 3.4%), left main PA (13; 3.4%), and right main PA (10; 2.6%). There were 137 (36.2%) stenosis, which were mainly located in the right lower lobe and its segmental stage and the right upper lobe and its segmental stage. Occlusion cases were mainly located in pulmonary segmental stage. Pulmonary trunk was involved in 16 patients at 20 locations (5.3%). Right-side PA was involved in 54 patients at 235 locations (62.2%). Left-side PA was involved in 36 patients at 123 locations (32.5%). The lesion of PA in the right was more frequent compared to the left. The right upper lobar PA was most frequently found in 81 (21.4%) instances. Among them, apical segment occlusion [20 (5.3%)] was the most frequent.

In our study, pulmonary artery damage was the most common damage in TAK patients with PAI, accounting for 247 (65.34%) instances. Fifty-two of the 65 patients had segmental involvement, 26 patients had more than 3 segments involved, 11 patients had more than 10 segments involved, and the patient with the largest number of involved segments had 18 segments involved. The number of pulmonary segments involved for each case is showed in Fig. 2B.

### Cluster analysis outcome

**Fig. 3:**
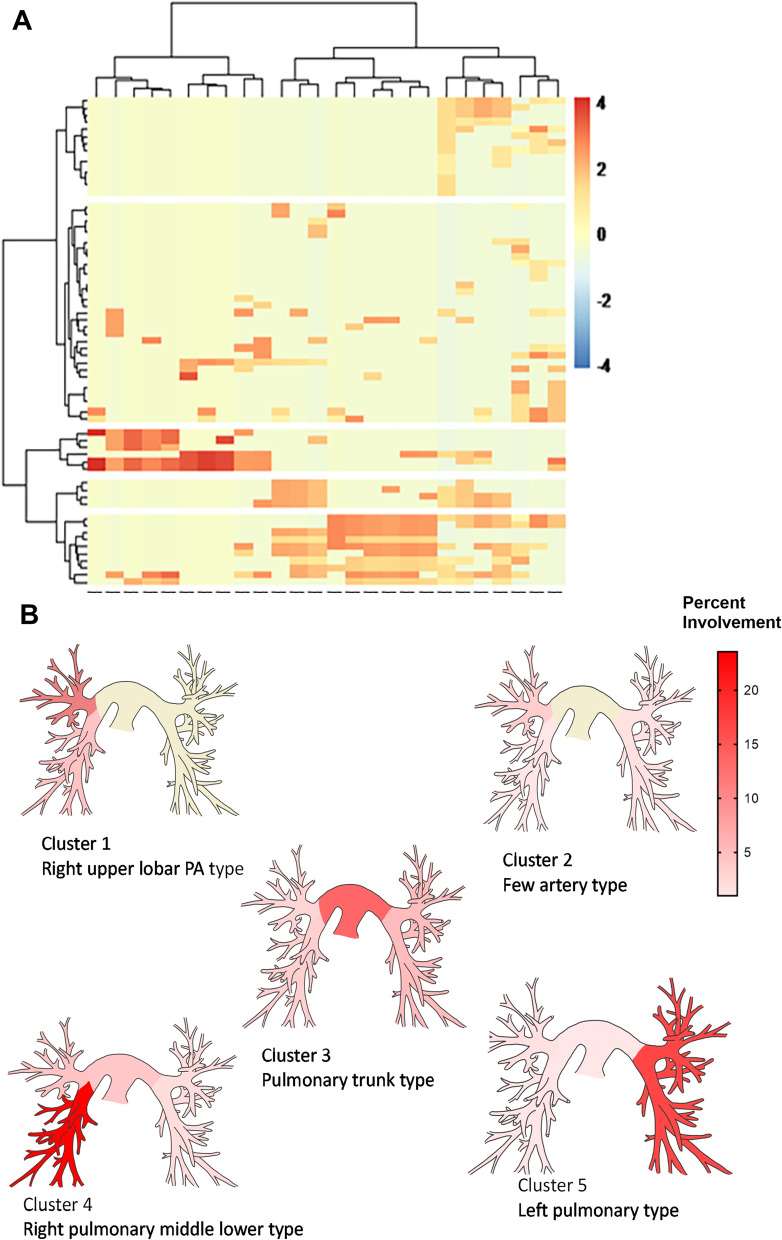
Clustering of the vascular involvement in the 65 patients with Takayasu arteritis pulmonary artery involvement (TAK-PAI). **A** The dendrogram on the bottom shows the clustering of the 26 vascular territories assessed in the cohort of 65 patients with TAK-PAI. The color map at the center indicates involvement (red) or noninvolvement (yellow) of the 26 vascular territories in each of the 65 patients. *X*-axis denotes different vessels and *Y*-axis denotes different patients. In the heatmap, negative four to four is the degree of vascular involvement and is normalized. **B** Prevalence of pulmonary artery involvement by cluster analysis. Heatmaps of the arteries indicated the percent of patients in each cluster with pulmonary artery involvement. A darker red indicates that a higher percentage of patients in the cluster have involvement of the pulmonary artery

Cluster analysis revealed five clusters of patients (Fig. 3; Table 4). Cluster1 was named as “right upper lobar PA type”. It was characterized by the involvement of the PA in the upper lobe of the right lung. Cluster1 had a higher frequency of fever (62.5% vs. 17.5%; *P* = 0.013), *Mycobacterium tuberculosis* infection (50% vs. 17.5%; *P* = 0.059), and hypoxemia (50% vs. 15.8%, *P* < 0.023) than non-Cluster1. Cluster2 was named as “few arteries type”. It was characterized by fewer involved PAs. In particular, left PA, right PA, and right lobar PA and its segment were rarely involved. Thirteen of the 19 patients had only one PA involved and six had two PAs involved. Cluster2 had a higher frequency of dizziness (68.4% vs. 37.0%; *P* = 0.021) than non-Cluster2. Cluster3 was named as “pulmonary trunk type”. It was characterized by the involvement of the main PA and left and right main PAs. Cluster3 had a higher frequency of PAH (30.4% vs. 16.7%; *P* = 0.329) than non-Cluster3, but this difference was not statistically significant. Cluster4 was named as “right pulmonary middle lower type”. It was characterized by the involvement of the right middle lobar PA and its segment and the right lower lobar PA and its segment. Cluster4 had a higher frequency of pulmonary infarction (75% vs. 35%; *P* = 0.076) than non-Cluster4, but this difference was not statistically significant. Cluster5 was named as “left pulmonary type”. It is characterized by the involvement of left PA and its lobar and segment.

**Table 4:** Angiographic features of clustering

Type	Name	*n*	Involvement of the pulmonary artery	Pulmonary trunk, Right main PA, Left main PA, *n* (%)				Right upper lobe and its segmental stage, *n* (%)					Right middle lobar PA and its segment, right lower lobar PA and its segment, *n* (%)				Left PA and its lobar and segment, *n* (%)			
				T	S	O	D	T	S	O	D	A	T	S	O	D	T	S	O	D
Cluster1	Right upper lobar PA type.	8	The upper lobe of the right lung	2 (3.45)	1 (1.72)	0 (0)	0 (0)	0 (0)	12 (20.7)	24 (41.4)	0 (0)	0 (0)	0 (0)	3 (5.17)	14 (24.1)	0 (0)	0 (0)	1 (1.72)	1 (1.72)	0 (0)
Cluster2	Few artery type.	19	Fewer number of involved pulmonary arteries	1 (4.17)	0 (0)	0 (0)	1 (4.17)	0 (0)	7 (29.2)	5 (20.8)	0 (0)	1 (4.17)	0 (0)	2 (8.33)	2 (8.33)	0 (0)	0 (0)	2 (8.33)	3 (12.5)	0 (0)
Cluster3	Pulmonary trunk type.	23	Main pulmonary artery	25 (27.2)	22 (23.9)	4 (4.35)	0 (0)	0 (0)	3 (3.26)	6 (6.52)	1 (1.09)	0 (0)	0 (0)	7 (7.61)	3 (3.26)	2 (2.17)	0 (0)	9 (9.78)	8 (8.70)	2 (2.17)
Cluster4	Right pulmonary middle lower type	8	Right middle lobar PA and its segment, right lower lobar PA and its segment.	7 (5.47)	8 (6.25)	0 (0)	0 (0)	0 (0)	0 (0)	17 (13.3)	0 (0)	0 (0)	10 (7.81)	30 (23.4)	49 (38.3)	0 (0)	0 (0)	2 (1.56)	5 (3.91)	0 (0)
Cluster5	Left pulmonary type	7	Left PA and its lobar and segment.	1 (1.31)	2 (2.63)	1 (1.31)	0 (0)	0 (0)	1 (1.31)	4 (5.26)	0 (0)	0 (0)	0 (0)	1 (1.31)	3 (3.95)	0 (0)	0 (0)	24 (31.6)	39 (51.3)	0 (0)

*T* thickening, *S* stenosis, *O* occlusion, *D* dilation, *A* aneurysm

### Cumulative incidence of disease progression events by the follow-up

One hundred forty-one TAK patients were followed up for a median of 46 (4–120) months. Sixty-five TAK patients with PAI were followed up for a median of 37 months (4–120), among whom, 42 cases did not experience any events, with a median follow-up duration of 53 (8–120) months. Twenty-seven events (1–3 per person) were observed in 23 (35.4%) patients, with a median follow-up duration of 18 months (4–66). Seventy-six TAK patients without PAI were followed up for a median of 49 months (8–116), among whom, 70 cases did not experience any events, with a medium follow-up duration of 48.5 months (8–116). Seven events (1-2 per person) were observed in 6 (7.9%) patients, with a median follow-up duration of 57.5 months (16–84).

CTPA confirmed that PA exacerbation or new PA involvement occurred in 26 patients including 1 patient who died and 25 patients who had disease progression events. Four patients died, which was attributed to all-cause mortality. Three of these were TAK patients with PAI. Among them, one died of exacerbation of PAH, one died of heart failure, and one died of side effect of the drug. The death of the TAK patient without PAI was due to exacerbation of TAK. In TAK patients with PAI, exacerbation of PAH occurred in 5 patients, exacerbation or new onset of hypoxemia occurred in 15 patients, and exacerbation of chest pain occurred in four patients. In TAK patients without PAI, new onset of hypoxemia occurred in four patients, new onset of chest pain occurred in two patients, and there was no occurrence of new pulmonary infarction.

**Fig. 4:**
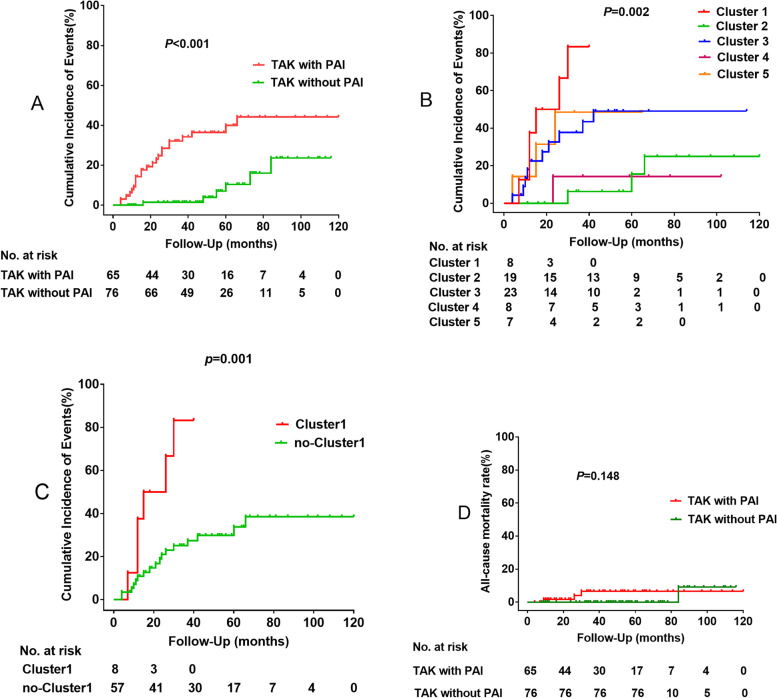
Cumulative incidence of events. **A** Cumulative incidence of events in TAK with PAI and TAK without PAI. **B** Cumulative incidence of events in five cluster. **C** Cumulative incidence of events in Cluster1 and non-Cluster1. **D** All-cause mortality rate in TAK with PAI and TAK without PAI

Kaplan-Meier curve revealed that TAK patients with PAI had a significantly higher cumulative incidence of events than those without PAI (*P* < 0.001) (Fig. 4A). Cumulative incidence of events was significantly different among the five clusters (*P* = 0.002). Cluster1 had the worst prognosis (Fig. 4B). There was no difference between Cluster3 and Cluster5 (*P* = 0.881). There was no difference between Cluster2 and Cluster4 (P = 0.900). Also, there was no difference in the cumulative incidence of events among Cluster2 and Cluster4 and TAK without PAI groups (*P* = 0.521). Patients with Cluster1 suffered from worse cumulative incidence of events in comparison with non-Cluster1 patients (*P* = 0.001) (Fig. 4C). There was no difference between TAK with PAI and TAK without PAI in all-cause mortality rate (*P* = 0.148) (Fig. 4D).

## Discussion

To the best of our knowledge, the present study is the first to identify that the involvement of the thoracic aorta in TAK patients is related to PAI. Also, this is the first time that the cluster analysis of PAI in TAK with PAI has been reported, where it was found that Cluster1 (named as “right upper lobar PA type”) had the worst prognosis.

Our results about the relationship between the involvement of the thoracic aorta in TAK patients and PAI suggest that PAI should be considered once thoracic aorta involvement is identified in TAK patients. Sporadic evidence pertaining to the relationship between PAI and the involvement of thoracic aorta can be found in the literature [8, 14–21]. In a study from Korea analyzing data of 12 TAK patients, the two patients who had PAI also showed the involvement of thoracic aorta [14]. In another study from Korea evaluating CT angiography of 10 TAK patients [15], the three patients who had PAI [22] were among those diagnosed with the involvement of aortic arch and its branches. In other case studies, PAI has also been reported to be accompanied with wall thickening of the thoracic aorta [8, 16–19], dilation or narrowing of the ascending aorta [20], and slightly prominent thoracic aorta [21]. Thus, the involvement of the thoracic aorta can be used as a predictor of PAI in TAK patients.

In our study, right upper lobar PA type (Cluster1) had the worst prognosis. A higher frequency of fever, worse hypoxemia status, and higher prevalence of *Mycobacterium tuberculosis* infection were observed in patients with Cluster1. In our study, Cluster2 and Cluster4 had good prognoses, with no difference in the incidence of the involved disease progression events compared with the non-PAI group. The prognosis of the few arteries type (Cluster2) and the right pulmonary middle lower type (Clsuter4) was good. In a few arteries type (Cluster2), only one or two PAs were involved and in the right pulmonary middle lower type (Clsuter4) right middle lobar PA and its segment, and right lower lobar PA and its segment were involved, which indicate good prognosis. The prognoses of pulmonary trunk type (Cluster3), which involved main PA, and left pulmonary type (Cluster5), which involved left PA and its lobar and segment, were worse than those of Cluster2 and Cluster 4, suggesting that patients with main PA or left PA involvement should be treated actively. Cluster1 had the worst prognosis, where the right upper PA and its branches were mainly involved. The patients of Cluster1 had a high incidence of *Mycobacterium tuberculosis* infection (TB), and the clinical symptoms of fever and hypoxemia were more frequent. Many studies have found that TB is common in patients with TAK [23–25]. Several research reports have attempted to explain how TB triggers TAK, but the exact mechanism in place for TB in TAK is still unclear. One possible mechanism is the cross-reactivity between mycobacterial antigens including the 65-kDa heat-shock protein (HSP65) and human 60-kDa HSP (hHSP60) [26–28]. Pulmonary tuberculosis is more likely to occur in the right lung because of the physiological structure of this area. More specifically, because the bronchus of the right lung is straight and larger, the chance of tracheal foreign body entering the right side is more common compared with the left side. The tuberculosis bacillus is an aerobic bacterium, meaning that its presence in the upper lung with less blood flow and high oxygen content is conducive to the implantation and growth of the binding bacillus. This leads to a situation, where the primary pulmonary tuberculosis is usually found in the upper lung. TB aggravates the process of systemic inflammation and Takayasu arteritis. Thus, the right upper lobar PA type (Cluster1) had the worst prognosis. Our findings suggested that patients with a history of *Mycobacterium tuberculosis* infection should be vigilant about the involvement of the right upper PA to guide the clinical treatment and prognosis.

Our study demonstrated that oppression in the chest and fever were more frequent in patients with TAK with PAI compared with those with TAK without PAI, which was consistent with a previous report by Kong et al. [29]. Furthermore, we found that hemoptysis was more common in TAK with PAI compared with TAK without PAI, which was in line with the findings of previous studies [7, 30]. We also found that TAK with PAI was more frequently accompanied with *Mycobacterium tuberculosis* infection than TAK without PAI. One study showed that among the TAK patients studied, about 17.7% had a history of tuberculosis, which was higher than that of the general population, which is 5.5–5.8% [23]. Another study showed that among the 1105 TAK patients considered, 109 (9.9%) had tuberculosis, where these patients showed more frequent involvement of the PA [24]. The exact mechanism and the relationship in place between TAK and *Mycobacterium tuberculosis* infection remain to be understood. A possible hypothesis is that *Mycobacterium tuberculosis* infection can promote the differentiation of CD4+ T cells into Th1 and Th17 lymphocytes [31–33]. We should add that we did not find an association between PAI and disease activity, which is in accordance with a previous study [29].

Many studies have found that PAH is more common in TAK with PAI. According to previous reports, the prevalence of PAH in TAK patients with PAI widely varied, in the range 15.74–61.7% [7, 29, 30, 34]. Our study also demonstrated that TAK patients with PAI had a higher risk of PAH, pulmonary infarction, and hypoxemia than TAK patients without PAI.

In previous studies, the incidence of PAI in patients with TAK who had undergone imaging of the PAI varied in the range 6.9–86% [7, 35–39]. In the present study, the prevalence of PAI was found to be 46.1%. Fifty-two of the 193 TAK patients in the study did not have CTPA. Therefore, the incidence of PAI in our center is not low. Our CTPA data showed that the lesion of PA in the right was more frequent than that in the left, and the right upper lobe PA was the most affected area. He et al. have reported that the right upper lobar branches were the most frequently involved regions (72, 56.3%). Our study showed that stenosis and occlusion were the most common presentation of PAI in TAK. This conclusion is consistent with the results of many studies [7, 29, 30, 40, 41]. Furthermore, in our study Numano type V was found to be the most common type (63.1%), which is in line with the findings of previous research [29, 30].

A longer disease duration was found in TAK with PAI, which is in line with the finding of a previous study [42]. According to prior reports, longer disease duration is associated with the progressive disease course [43, 44], where progressive disease course has been identified as an independent predictor of mortality [37, 45], or has been significantly associated with higher mortality [43]. This suggests that PAI in TAK is related to higher mortality. It should be added that in one study [34] no difference was found in mortality of TAK patients with and without PAI, which was attributed to the small sample size by the authors.

TAK patients with PAI have longer disease duration; have higher cumulative incidence of events; have higher risks of pulmonary hypertension, pulmonary infarction, and hypoxemia; and more frequently have hemoptysis, oppression in the chest, fever, and *Mycobacterium tuberculosis* infection than TAK patients without PAI. In TAK, PAI is associated with oppression in the chest and thoracic aorta involvement. In TAK patients with PAI, the involvement of the upper lobe of the right lung is characterized with the worst prognosis.

Our study has some limitations. First, it has a retrospective design. Second, the sample size is small. A prospective cohort study with a larger number of TAK cases is needed to confirm the characteristics of the pulmonary involvement in TAK patients.

## Conclusion

In conclusion, this study demonstrated that the involvement of the thoracic aorta in TAK patients is related to PAI. Also, this study reports for the first time a cluster analysis of PAI in TAK with PAI, where it was found that the right upper lobar PA type (Cluster1) had the worst prognosis.

## Abbreviations

TAK: Takayasu arteritis
PAI: Pulmonary artery involvement
PA: Pulmonary artery
PAH: Pulmonary arterial hypertension
CTPA: Computer tomography pulmonary angiography
NIH: National Institutes of Health
CTA: Computed tomography angiography
MRA: Magnetic resonance angiography
OR: Odds ratio
CI: Confidence interval
WBC: White blood cell
HGB: Hemoglobin
PLT: Platelet count
ESR: Erythrocyte sedimentation rate
CRP: C-reactive protein
T: Thickening
S: Stenosis
O: Occlusion
D: Dilation
A: Aneurysm
RUL: Right upper lobe
RML: Right middle lobe
RLL: Right lower lobe
LUL: Left upper lobe
LLL: Left lower lobe
